# ECO6G: Energy and Cost Analysis for Network Slicing Deployment in Beyond 5G Networks

**DOI:** 10.3390/s22228614

**Published:** 2022-11-08

**Authors:** Anurag Thantharate, Ankita Vijay Tondwalkar, Cory Beard, Andres Kwasinski

**Affiliations:** 1 School of Science and Engineering, University of Missouri, Kansas City, MO 64110, USA; 2 Department of Computer Engineering, Rochester Institute of Technology, Rochester, NY 14623, USA

**Keywords:** Beyond 5G, energy efficiency, machine learning, network load, network slicing, OPEX savings

## Abstract

Fifth-generation (5G) wireless technology promises to be the critical enabler of use cases far beyond smartphones and other connected devices. This next-generation 5G wireless standard represents the changing face of connectivity by enabling elevated levels of automation through continuous optimization of several Key Performance Indicators (KPIs) such as latency, reliability, connection density, and energy efficiency. Mobile Network Operators (MNOs) must promote and implement innovative technologies and solutions to reduce network energy consumption while delivering high-speed and low-latency services to deploy energy-efficient 5G networks with a reduced carbon footprint. This research evaluates an energy-saving method using data-driven learning through load estimation for Beyond 5G (B5G) networks. The proposed ‘ECO6G’ model utilizes a supervised Machine Learning (ML) approach for forecasting traffic load and uses the estimated load to evaluate the energy efficiency and OPEX savings. The simulation results demonstrate a comparative analysis between the traditional time-series forecasting methods and the proposed ML model that utilizes learned parameters. Our ECO6G dataset is captured from measurements on a real-world operational 5G base station (BS). We showcase simulations using our ECO6G model for a given dataset and demonstrate that the proposed ECO6G model is accurate within $4.3 million over 100,000 BSs over 5 years compared to three other models that would increase OPEX cost from $370 million to $1.87 billion during varying network load scenarios against other data-driven and statistical learning models.

## 1. Introduction

The 5G mobile communication network serves as a communication infrastructure that converges connectivity, intelligent edge, and the Internet of Things (IoT) from consumers to industries. As such, 5G is revolutionizing businesses and society by enabling high-speed broadband with ultra-low latency, high capacity, massive connectivity, and reliability. To achieve sustainable development goals and create an environmentally conscious infrastructure to improve people’s living standards, it is of utmost importance that the 5G network provides high speed and reduced latency with significantly lower network energy consumption. The 5G standard enables the MNO to optimize the Quality of Service (QoS) and improve the Quality of Experience (QoE) for end-users with the help of KPI metrics such as network load, battery level, and signal strength. These 5G KPIs then guarantee both network and device efficiency, which has always been the fundamental concern for MNOs and device manufacturers from an optimization standpoint. When combined with ML, 5G can further help grow businesses efficiently and grant consumers access to more information faster than ever. On the path to the future generation networks, we must develop an AI/ML-defined network infrastructure that is energy efficient and can learn from its dynamic environments [1].

Overall, 5G is an inherently greener technology with more data bits per kilowatt (kW) energy than previous generations of wireless technology. However, the exponential growth in data traffic necessitates additional Energy Efficiency (EE) and Carbon dioxide (CO_2_) reduction measures. The Global System for Mobile Communications Association (GSMA) found that the 5G data traffic has grown exponentially since its commercialization. By 2025, it is anticipated that the 5G data traffic will be eight times higher than fourth-generation (4G)/Long-Term Evolution (LTE), and twelve billion devices will be connected to the 5G and IoT. These subscribers are expected to consume 5–10 times more than 4G (LTE) subscribers. The MNOs will need more ways to keep network energy consumption low as 5G services mature. According to the GSMA Intelligence Report, 67% of mobile service providers anticipate rising energy expenditures. Although 5G is more energy efficient, increasing traffic demand and complicated use cases will increase the total energy consumption. On the positive side, the mobile industry has collaborated to build a climate action plan to attain net-zero greenhouse gas emissions by 2050, with over 30% of carriers making public commitments. The MNOs plan to optimize 5G networks for EE to reduce their carbon footprints and energy using ML models to improve traffic prediction accuracy [2,3].

Developing 5G optimization strategies for EE that address data processing capacity and latency concerns is critical, especially for network slicing in the 5G architecture. Slicing a network refers to the process by which a network operator divides a single physical network into logically distinct networks. Networks are established to provide specialized networks for diverse service providers with varying characteristics. Currently, the third-generation partnership project (3GPP) has defined three network slices: enhanced Mobile Broadband (eMBB), massive Machine-type Communication (mMTC), and Ultra-Reliable Low-Latency Communication (URLLC). To efficiently deliver these tailored services with varying KPI requirements, operators must employ more integrated and sophisticated methods than they did in 4G. Additionally, 5G’s cloud-based architecture, which enables greater scalability and elasticity, is a significant differentiator from its predecessor, which enables operators to deploy new network functions (NFs) without incurring the additional Capital Expenditure (CAPEX) to meet demand better [4].

For decades, the MNOs have prioritized throughput, coverage, and data latency for building networks. However, due to environmental and economic concerns, network energy efficiency has recently emerged as a significant factor for next-generation network deployments. With the advent of high-capacity traffic services, wireless data traffic has increased exponentially; this increase in wireless data traffic degrades the existing network efficiency. To maintain QoS and per-bit cost, network operators must increase data traffic exponentially. Focusing on high-data-rate services increases the network’s energy consumption, posing environmental and financial concerns. Hence, in modern wireless network operation and design, the MNOs must consider EE as one of the KPIs due to environmental, economic, and operational concerns [5].

## 2. Contribution

Our primary contributions in this research work are as follows:

- We have discussed the motivation for analyzing the EE using ML approaches and the challenges in current 5G and Beyond networks in Section 4 and Section 5.
- We have evaluated our proposed ECO6G model against the traditional deep learning neural network (DLNN) with random weights and statistical time-series modeling, i.e., Auto-Regressive Integrated Moving Average (ARIMA) and Exponential Smoothing (ETS) in Section 6.
- We have modeled the CAPEX for an MNO according to the EE definitions defined in ETSI (European Telecommunications Standards Institute) TR 132 972 [6,7] and highlighted the ECO6G load prediction usefulness towards the OPEX saving for MNOs in Section 9.

## 3. Related Work

The 3GPP Release 17 [8] work item has limited use cases, requirements, and solutions for measuring the EE of 5G networks, including Next-Generation RAN, core network, and network slices, for optimizing the EE or managing energy savings in 5G. Energy-efficient KPIs have been defined for network slices, including eMBB, URLLC, and IoT. However, V2X remains unaddressed. Additionally, there is no definition for the URLLC network slice reliability EE KPI in 3GPP definitions today. DeepSlice [9], and Secure5G [10] studied the network slices in 5G systems by applying DNN techniques. We have demonstrated network slice selection for all UE types, including unknown devices, load-balancing techniques in case of slice failure, and security of these slices in case of a DDoS attack. We have used various KPIs, such as the 5G QoS Identifier (5QI), Packet loss rate, Packet Delay budget, UE types, Day, and time, to simulate the models. In Table 1 we have done the comparison of ECO6G against state-of-the-art methodologies.

Network function virtualization (NFV) enables the deployment of such network slices on general-purpose servers. With the help of NFV, network slicing can customize virtual network functions (VNFs) to adhere to different customer services. However, due to the complex architecture, VNF are vulnerable to the anomaly of physical nodes (PN) from the shared substrate networks. The Hidden Markov model (HMM) is a practical approach to deducing the anonymous network information (working state of the PN) through observing VNFs measurements (observable outputs) mapped to it. The learning time for a HMM is prolonged. To accelerate the time required to converge to the optimal solution, the paper [22] proposes a cooperative anomaly detection scheme by employing the TL-based hidden Markov model (TLHMM) and validates the algorithm’s efficiency under different network configurations.

The authors in [23] address the potential challenges in adapting to the non-stationary wireless environment and propose solutions for using AI to manage resources for the B5G network slicing. Additionally, the work targets the impact and issues for resource management in network slicing when a Non-Terrestrial Network is integrated into B5G systems. Networks can support multiple services, with each service demanding a different KPI. Hence, it is vital to efficiently assign computing and network resources, considering all the KPIs (including availability and reliability) targeted by a service. The authors in [24] propose a resource allocation scheme OKpi that facilitates high-quality selection of radio points of access and VNF placement and data routing with polynomial computational complexity. The convergence of graph theory and optimization (OKpi) yields reliable performance in evaluating the two real-world scenarios described. Unlike [23,24], our ECO6G framework can enable slice-level analytics and provide either statistics or forecasts regarding the performance of network load, which can be further used to design and orchestrate network resources.

The paper [25] investigates the problem of energy consumption and network load imbalance in RANs, using load prediction to estimate the traffic load. It highlights two approaches: one with a linear ensemble model, a combination of seasonal ARIMA (SARIMA), linear regression, and regressive trees that learns the traffic features from three different perspectives: time, space, and historical pattern. The results from the SARIMA are evaluated using Average Normalized RMSE (ANRMSE) as the evaluation index of performance. In contrast, the second approach uses a ResNet that uses traffic’s spatial and temporal dependencies to estimate the traffic load accurately. Our work follows a similar approach of comparative analysis of the ECO6G model with ARIMA, ETS, and DNN model with random weights to predict the load estimation and use it to analyze the network’s overall efficiency.

The paper [26] analyzes energy consumption management, which is fundamental to the deployment of network slicing in 5G networks. Unlike other literature surveys, the paper presents an economical solution for NFV, SDN, and green energy attributes of network slicing that can potentially contribute to reducing OPEX and environmental impact, thereby improving service provisioning. Our approach to reducing energy consumption (increasing energy efficiency) and increasing the OPEX savings of the MNO is employing a DNN model with TL to accelerate learning and achieve convergence. The heterogeneous deployment of BSs and APs in 5G slice networks facilitates meeting the QoS requirements for different network use cases. However, the unplanned deployment may increase the total energy consumption. The paper [27] proposes an energy-efficient resource allocation algorithm for network slicing (EERAN), which offers low complexity and faster convergence over extensive Monte Carlo numerical simulations.

With the exponential evolution in wireless mobile communications and related technological advances, the MNOs are required to invest in network infrastructures such that the CAPEX and OPEX are balanced to provide network services with acceptable QoE. The software-defined networks (SDNs) and the cloud radio access network (C-RAN) can be the enabling technologies for this challenge. The paper [28] proposes a C-RAN framework based on SDN that can be efficiently operated by the MNOs. The authors propose an ANN-based load balancing and mapping algorithm, BBU-RRH (baseband units- remote radio head). Wireless sensor networks (WSNs) are considered an integral part of the design of IoTs. Therefore, even Industry 4.0 encompasses the interconnectivity of such sensor nodes, IIoT (Industrial IoT), and intelligent manufacturing. However, the main challenge in designing the WSNs is network slicing and data aggregation. The paper [29] proposes an energy-efficient DL-based network slicing with a data aggregation (EENS-DA) approach that efficiently allocates physical resources in resource-constrained networks. The paper [30] proposes a DL-based load forecasting for smart grids. The load forecasting performance of CNN (Convolutional Neural Network) was compared against ANN, SVM (Support Vector Machine), LSTM, LSTM-S2S, and Factored Conditional Restricted Boltzmann Machines (FCBRM) in terms of RMSE as the evaluation metric of performance.

The paper [31] proposes a computationally efficient, scalable, priority-based algorithm for WSNs referred to as the most energy-efficient resource first (MEERF). The MEERF is compared against the benchmark policies Maximal Transmission Rate (MTR) and the Minimal Power Consumption Policy (MPC), and the MEERF outperforms the other policies regarding energy efficiency. The paper [32] addresses the OPEX limited resource provisioning for network slicing in 5G cellular systems. Network slicing increases statistical multiplexing (sharing the same physical resources as slices for different applications). It is essential to ensure a flexible and dynamic resource allocation, and in this context, Deep Neural Networks, DNNs can be used for end-to-end resource provisioning. The problem is formulated by optimizing the network slicing OPEX with resource allocation. The paper [33] uses ML to facilitate energy-efficient future 5G networks towards a complete self-organized network (SON). The ML approach is implemented to analyze the real-time network-generated data (cell-level traces of an LTE network) to predict the future state of the network promptly. Since network slicing is an enabler of different services in 5G networks, ref. [21] proposes a DL based network slicing short-term traffic prediction and proactive adjustment framework. The network slice traffic is accurately predicted using gated recurrent units, following which the network slices are dynamically adjusted. The framework demonstrates an improvement in the network utilization efficiency of the 5G transport network.

The paper [34] addresses the problem of decision making for network management and reducing slicing failures by putting forth a hybrid DL model. The CNN in combination with LSTM achieves an overall accuracy of 95.17%, where the CNN is responsible for resource allocation and slice selection and LSTM manages the load balancing of the network slices. The need for efficient network slicing in 5G is important from the perspective of reducing the network operator’s cost energy consumption while providing quality of service. The paper [35] addresses this problem by modeling a joint optimization problem of energy consumption and cost, alongside a prediction-assisted dynamic network slice algorithm for adaptive network slice allocation in accordance with the service requirements. The paper [36] proposes a 5G micro-service-based prototype which facilitates allocation of resources for network slices. This allocation is powered by ML decisions based on the KPIs from real-time data. Even though the data-driven approach increases the throughput of the network, it increases the resource utilization.

The authors [37] propose a ML-based network sub slicing architecture for IoT applications to optimize the network load balancing problem, at the same time maintaining latency, heterogeneity and power efficiency using SVM and K-means for feature selection. The authors [38] investigate and propose a framework for the network slicing technology as an enabler of different use cases in 5G. The paper also highlights the challenges associated with Network as a Service (NaaS) and future research opportunities. The paper [39] targets the use case of emergency services and communications for 5G network slicing by proposing a RESPOND-A platform that provides first responders with network-enabled tools above 5G on-scene planning specifically targeted at emergency-related communications. The work [40] puts forth a DL and block chain based security framework that ensures scalability, reliability, performance, security, and privacy of a 5G enabled IoT environment. The authors [41] investigate the role of wearable computing for defense automation system and thereby propose a prototype design to a covert and efficient communication in 5G. The work in [42] puts together a comprehensive survey of emerging 5G technologies, such as the Block chain, D2D communication, SDN, AI and mobile edge computing, as well as the issues and solutions associated with 5G security. The paper also highlights various applications and use cases of 5G security, such as automotive driving, IoT services, drones, etc.

## 4. Motivation for Energy Efficiency Using Data-Driven Learning

Energy consumption comprises a considerable portion of network OPEX, and BSs are the radio access network’s primary energy-consuming equipment. To achieve Radio Access Network (RAN) EE, turning off cells during off-peak hours is one way to reduce network energy usage; it would be ideal if the MNO could estimate the future load efficiently and configure resources accordingly. Predicting a network function overload or outage enables operators to take preventative measures (for example, to avoid selecting a heavily loaded node for a latency-sensitive/resource-intensive service) to ensure smooth network operation and improve the 5G customer QoE. Other techniques for improving EE include adjusting a BS’s coverage area based on its load level, favoring lightly loaded BSs to sleep, and load balancing by handing over the User Equipment (UE) to the micro or pico base station.

In contrast, network operators continue to deploy 5G and employ novel New Radio (NR) features such as beam forming, dynamic spectrum sharing, multiple-input multiple-output (MIMO), and network slicing, introducing complex system design and optimization challenges. The MNOs struggle with traditional hard-coded algorithms, which require human–machine interaction, which is error prone, slow, costly, and cumbersome. Artificial intelligence (AI), including ML algorithms, can help operators improve network management and user experience by analyzing and processing network KPIs and metrics. AI in 5G networks has captivated academia and industry to explore optimization methods for UE trajectory prediction, traffic steering, load balancing, energy saving, and massive MIMO configuration optimization [43]. AI and ML are enabling operators to gain new capabilities and efficiency gains. They enable network equipment to sense, reason, infer, and bring novel solutions to technological issues. A holistic and end-to-end approach to AI and ML can provide a pervasive system-level approach to energy efficiency improvements spanning hardware, software, and algorithms. Energy management is a data-intensive operation; without AI, operators cannot efficiently process information and make real-time choices at scale. To implement adaptive energy management in the network slicing, the MNOs can assign different priority levels to differentiate services between slices, such as emergency services or service characteristics (e.g., number of end-users, location, average consumption) [26].

Energy consumption is a significant issue, both environmentally (carbon footprint) and economically. The energy consumption of the 5G BSs is so high that electricity bills have become one of the most significant expenses for 5G providers. Costs to the MNOs are expected to increase significantly over the next five years. Studies suggest that, on average, MNOs spend 25 billion dollars annually on energy. Telco industry reports suggest EE and optimization are crucial for network transformation and climate action agendas. Energy is the only significant operational expense predicted to rise soon [4]. The 5G BS energy savings involve hardware and software, multiple power-saving features, small cell deployments, and new 5G architecture and protocols that can be combined to improve wireless network energy efficiency. Optimizing hardware architecture, production process, and integration of crucial core chips such as base-band processing, digital intermediate frequency, and radio-frequency modules reduces hardware energy consumption on AAU (Active Antenna Unit) and RRU (Remote Radio Unit).

Low-traffic areas account for 70% of network sites in most cases, but carry only 25% of the total traffic. Only 30% of network sites are in medium to high traffic areas, yet they carry 75% of all traffic. Historically, the industry has prioritized high-traffic sites and neglected low-traffic networks. This provides a massive opportunity for MNOs to use predicted load as one element to design network and energy optimization strategies [2], where BS resources must be scheduled according to service load to conserve energy. In [44], the authors have compared the load forecast for a single cell using several prediction techniques. The simulation results of load prediction are based on the consumption of fifty physical resource blocks (PRBs). Compared to each standalone sub-model, the ensemble learning model has significantly enhanced the accuracy of its predictions. The developed ensemble learning method reduces the average Mean Absolute Error (MAE) by 0.008. The load prediction models include Arima, Prophet, Random Forest (RF), Long Short-Term Memory (LSTM), and Ensemble learning. The models use historical and current loads to predict future loads, so historical traffic loads are considered when building and training the ML model.

Because ML models can swiftly analyze substantial amounts of data from many sources, they improve the potential for network-wide energy savings. AI algorithms can be optimized to assess real-time demand, traffic patterns, and network resource availability, and translate these data into actionable insights. In that case, more efficient resource management and network planning can be achieved, which is the primary motivation for this study.

## 5. Current Energy and Power Challenges in Beyond 5G Networks

Power saving has been a challenge since the second generation (2G) of wireless communication. The massive MIMO and high output power needs of 5G have worsened this issue. Massive MIMO and high output power requirements to service the increasing number of connections and data traffic will further raise energy demands. Running redundant network resources ensures excellent network availability, even if other resources fail, but wastes a great deal of energy. Network traffic varies by time and place, so different elements of the RAN infrastructure in each area can be put to sleep for predetermined periods. The more components of that BS that are turned off, the more energy is saved. There is an opportunity to develop more profound and extended sleep periods when no or fewer data transmissions occur, lowering the overall network energy consumption.

Currently, industries are experimenting with AI-powered solutions for simple operations such as shutdown and sleep cycles for cells serving users based on the modeled estimated traffic patterns modeled. These models are built on historical patterns, weather, local events, and other variables that can save energy by turning off power amplifiers, transceivers, and antennas. Such solutions can also help with load balancing, intelligent beam forming, interference reduction, and better spectrum utilization, among other things. In cellular networks, BSs consume the most power; studies show that BSs consume between 60 and 80% of all cellular network power, even when not serving any users. The increasing traffic demand and complicated new 5G use cases mean that 5G consumes more energy than earlier wireless technologies. Thus, putting a BS to sleep or turning it off entirely when there is little user traffic can help reduce cellular network power consumption. Additionally, BS experiments are application layer friendly and do not necessitate network changes and standardization, making them less costly and easier to evaluate and implement [45].

As studied in [46], most network expenses are attributable to energy consumption (fuel and electricity). BS sites are the primary energy consumers in a mobile network, requiring around 73% of a typical operator’s total energy in 2021, according to a GSMA analysis of thirty-one MNOs. RAN energy consumption comprises the eNodeB (4G BS), gNodeB (5G BS), as well as the energy consumption of associated equipment, such as air conditioning (AC), inverters, and rectifiers. The core network energy consumption comes from the network operations centers, value-added service platforms, and any energy consumption connected with backhaul transport. Furthermore, the energy spent by data centers includes the physical locations that host the infrastructure of operators, including Operational Support Systems (OSS) and Business Support Systems (BSS). It is important to note that the AC is still running and consuming the same amount of power, even when the network has low and medium load scenarios and other associated equipment.

The current NR design supports basic energy-saving measures, such as a gNB that can turn capacity cells on/off to save energy. The gNB autonomously makes decisions without knowing the impact on neighboring nodes or the overall network energy consumption. When neighboring nodes make conflicting decisions, the situation worsens. Additionally, the current energy-saving tools are limited to cell deactivation. With NR beam forming and multi-layered radio transmission structure, reducing the load in a coverage area or modifying the configuration of RAN nodes for coverage and capacity can reduce energy consumption. The optimal EE decision is conditional on many variables, such as node load, RAN node capabilities, KPI/QoS requirements, active UEs and mobility, and cell utilization; hence, optimizing EE at the RAN level using pre-defined and hard-coded rules is difficult.

ML can maximize the EE of a network by collecting pertinent data and taking the appropriate action. Utilizing a solution at the RAN level can reduce network energy consumption while maintaining coverage, capacity, and QoS. The ML model could use internal node information, neighboring RAN node information, and UE assistance information to make an EE determination (such as offloading UEs, deactivating/activating capacity cells, and adjusting node configuration) and communicate it to neighboring nodes. Neighboring nodes can provide feedback on the EE of a decision, and UE may also indicate if performance requirements are not met, indicating that the network should modify EE. The potential for an ML-assisted solution can be enhanced by exchanging RAN-level metrics for energy savings/consumption. MNOs can introduce an energy status that can be communicated between RAN nodes. Such indicators can assist neighboring nodes in understanding a node’s energy efficiency preferences, which can be considered when deciding on EE actions that may affect network energy consumption.

When a BS is powered on, its power consumption is proportional to the traffic volume. Research [44] demonstrates that around 60% of a BSs radio power usage scales with traffic load. When the predicted traffic volume is below the threshold, the cells can be turned off, and the UEs can be moved to the new target cell. ML algorithms can train the relevant model and predict the next period’s state, especially traffic load. In Rel-16, a new mechanism for exchanging the current load status of RAN nodes was added, which is used as input for Mobility Load Balancing (MLB)/Energy-saving algorithms. Additionally, based on our study and analysis, we believe that considering the predicted load status is beneficial, particularly for cells whose load status varies rapidly and follows a consistent pattern each day, especially in the case of network slicing, where logical networks can be managed independently.

Network Equipment Manufacturer reports [47] that compared to 4G, the power consumption per unit of traffic (Watt/bit) is drastically reduced, whereas 5G’s power consumption increases. The percentage of sites with more than five frequency bands will rise from 3% in 2016 to approximately 43% in 2023. Report shows that the maximum power consumption of a 5G site will be greater than 10 kW and will be doubled if more than ten frequency bands are used. A typical 5G site consumes more than 11.5 kilowatts of power, around 70% more than a BS that uses a mix of 2G, 3G, and 4G radios. The Network Equipment manufacturer forecasts that Massive MIMO alone can raise cell energy usage from 5–7 kW per 4G site per month to more than 20 kW per 5G site. China’s 5G energy usage is projected to increase by 488% by 2035, reaching 297 billion kWh [48].

## 6. Proposed ECO6G Framework

The 5G NR standard was developed with an understanding of typical radio network traffic and the requirement for radio network equipment to support sleep states. The BS can be put to sleep when no traffic is present in order to conserve energy. Even in heavily loaded networks, BS resources are often unused. Most base transceiver station (BTS) hardware components remain active to transmit 4G or 5G mandatory idle mode signals such as synchronization, reference, and system information [42]. The MNOs expect B5G deployment solutions to be low cost and capable of fast deployment, with low energy, and simple operations and maintenance to improve carrier investment efficiency. To mitigate these challenges, we propose an energy optimization method using the learning from the predicted load, which we have simulated using DNN, Transfer Learning (TL), ARIMA, and ETS models. The proposed ECO6G model is based on TL concepts, which utilizes a pre-trained model M_DNN, trained on a larger traditional DNN model.

The performance of any ML or DL model depends on size, quality, and relevance of the training dataset. Real-world datasets are disorganized and unstructured. Finding a balanced dataset or working with an imbalanced dataset is difficult, especially with the lack of field data for network slicing, which is not yet deployed in the production network. We believe that the field data is necessary for the ML model to function in real-world environments and for training, validation, and testing to ensure the validity and robustness of the model. Our ECO6G dataset is developed from a real-world 5G BS’s measurements using MNOs proprietary software, which includes data from one BS with three sectors and KPIs such as RRC, number of PDU sessions, and the total network load [49]. The dataset was collected over 52 weeks, of which 47 weeks were used for training, and the remaining 5 weeks were used for testing and validation. As network operators have yet to deploy network slices, we do not have the availability of actual slicing data. We have used the 3GPP specification TS 28.554 [50] definitions to augment the data for network slicing KPIs such as RSSNI and PDU session counts.

Traditional statistical methods use linear processing, whereas ML methods use non-linear algorithms to achieve minimization objectives. This paper employs four primary approaches to achieve time-series forecasting methods and comparisons: ARIMA, ETS, DL model using random weights, and a DL model using learned weights. The most challenging aspect of time series problems is that they predict an uncertain future. Predictions are never accurate and are always subject to variance, and it is challenging to discover and learn underlying patterns in time series data. Typically, patterns are categorized as trends, seasonality, and cycles. In most time-series data, these patterns are strongly interconnected, and it is difficult to distinguish and locate them due to short data length, noise and outliers. In the past, univariate time-series analysis and prediction problems were primarily addressed; however, multiple time-series data have gained prominence in recent years. We have performed a comparative study between ML and statistical modeling to rule out any issues with model superiority.

ECO6G utilizes TL, where weights are learned from a traditional DNN model $M_{DNN}$ trained on a larger dataset comprising the three KPIs from each slices and the total network load from one BS. The knowledge transfer in the case of TL eliminates the need to train an ECO6G model from scratch, resulting in faster convergence using a smaller training sample size. Finding sufficient and high-quality training data is one of the most challenging tasks for conventional ML techniques. By leveraging the trained knowledge from similar domains with high-quality data, TL can circumvent this issue. Instead of learning from scratch, as with conventional ML approaches, the training process for ECO6G can be significantly accelerated by incorporating knowledge from an $M_{DNN}$ model. Instead of maximizing the QoS, we argued that better EE could be achieved by targeting satisfactory QoS levels. Furthermore, accurate prediction of estimated network load based using recent (more real-time) data, which is also smaller in size, can be used for predictive analytics.

## 7. Process Flow for ECO6G Framework

**Figure 1:**
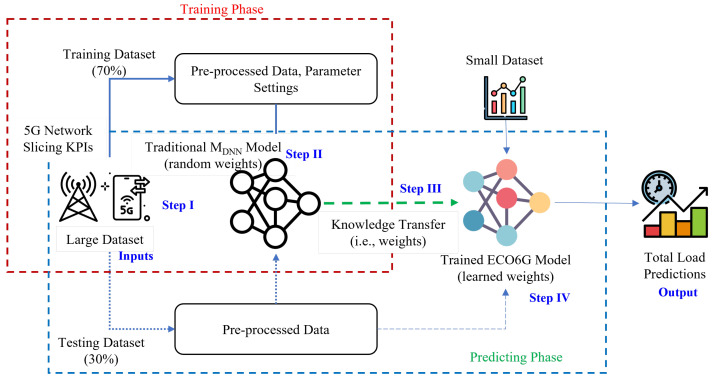
ECO6G Framework.

In this section, we detail the working of the proposed ECO6G, as shown in Figure 1:

Step I The ECO6G framework initializes by training the traditional neural model $M_{DNN}$ using observed RRC, number of PDU sessions, and the total network load from all Slices—A (eMBB), B (mIoT), and C (URLLC), i.e., $D_{TOTAL}$. Network operators can deploy many slices; we are considering three standard slices for our evaluation per standardized 3GPP SST values. We have employed five-layer DNN: Input (features), three Hidden Layers, and Output (prediction). We have tuned the model hyper-parameters by changing the number of hidden layers, learning rate, activation function, and the number of epochs for the $M_{DNN}$ model in MATLAB using Deep Learning Toolbox and Alteryx Analytics Automation tool running on Intel hardware and Windows 11 operating system. Our goal is to validate the model performance between random weights and learned weights, so we kept the DNN modeling the same for both $M_{DNN}$ and $M_{ECO6G}$. The algorithm uses randomness to find a good set of weights for the specific input–output mapping function of the data, such that each time the training algorithm is run, a different network with a different model is fitted. The shuffling of the training dataset before each epoch also uses randomness, resulting in differences in the gradient estimate for each batch.
Step II First, we train the $M_{DNN}$ multi-layer model using a feed-forward backpropagation network with initialized random weights (stochastic gradient descent). A forward pass through the network is accomplished by iteratively computing each neuron in the subsequent layer until the output is achieved. We evaluate the output quality based on a cost function *C* and the desired result in the output layer. Mean squared error (MSE) is used as a loss function for evaluation.
Step III A backward pass is then used to optimize the cost function *C* after the first result has been obtained by readjusting the weights and biases. We aim to optimize the output by adjusting the entire neural network. Based on this, we can calculate the total loss and determine the model’s suitability (good or bad), and here, weights are adjusted to achieve a minimum loss. After backpropagation, we capture each layer’s computed weights (learned weights) for TL parameters and define these trained weights as $M_{ECO6G}$.
Step IV Now, training the $M_{ECO6G}$ with ‘random weights’, we initialize $M_{ECO6G}$ using learned weights and re-train for smaller datasets $D_{eMBB}$, $D_{mIoT}$, $D_{URLLC}$ from individual slices, which are subsets of $D_{TOTAL}$ to predict total network load.

In ECO6G, we are capturing weights on the final layer (i.e., the output layer); however, we can capture weights in the initial layer and middle layer, as referenced in [51]. The model’s performance depends on the neural network architecture, the change in neurons, and the hidden layer, which influences the model performance and energy consumption. The more time the model takes to converge, the more energy it consumes. The complexity of a NN-based algorithm primarily depends on the number of nodes in each NN layer, total training examples, M and number of epochs, N. The time complexity T of the ECO6G algorithm can therefore be approximated as:

(1) T=O(M∗N∗#nodesinlayer(i)∗#nodesinlayer(i−1))

ECO6G model pseudo-code can be written as Algorithm 1:

**Algorithm 1** ECO6G Training and Validation
**1**: Set parameters
**2**: θ∈ (0, 1): weights/parameters
**3**: b ∈ (0, 1): bias
**4**: α∈ (0, 1): learning rate to control change in θ and *b*
**5**: σ∈ (0, 1): sigmoid activation function
**6**: $\sum =x_{i}\theta_{i}$ where $x_{i}$ is the input, $D_{train}$ consisting of RRC, RSSNSI and PDU of each of the three slices from the network and devices
**7**: Weighted sum value $z=x_{i}\theta_{i}+b$
**8**: $D_{train}\leftarrow$ Training data for the network load of size 7729 X 9
**9**: $D_{val}\leftarrow$ Training data for the network load of size 169 X 9
**10**: Initialization of the multi-layer model, $M_{DNN}$ consisting of parameters θ in [0, 1]
**11**: Training of $M_{DNN}$ with $D_{train}$
**12**: Predicting the network load with error function $MSE_{i}=\frac{1}{n}\sum_{i=1}^{n}{\left(y_{actual}-y_{predicted_{i}}\right)}^{2}$ $MAPE_{i}=\frac{1}{n}\sum_{i=1}^{n}|\frac{y_{actual}-y_{predicted_{i}}}{y_{actual}}|$
**13**: Optimization of cost function J(θ) through back propagation and gradient descent with J(θ) in step 12 until convergence
**14**: Selection of the learned parameters $\left(\theta^{\left(1\right)},\theta^{\left(2\right)},\theta^{\left(N\right)}\right)$ representing the pre-trained model as $M_{pretrained}$
**15**: Using the $M_{pretrained}$ parameters for validating $D_{val}$ where $D_{val}\in D_{Total}$


## 8. ECO6G Framework Evaluation

With TL, most data are trained by other source domains before transferring the trained models to the target domain, reducing the computing requirements for target domain training. This is useful for wireless devices with hardware constraints, such as smartphones, IoT, and edge devices. Additionally, only knowledge, such as model weights and biases, must be transferred, reducing communication overhead [52]. Consequently, this can significantly improve the learning rate, which is especially important for developing applications with ultra-low latency for future wireless networks. Conventional ML training is computationally intensive. ECO6G uses all the layers of a pre-trained $M_{DNN}$ model for initialization; this strategy is anticipated to be advantageous because the initial layers capture more typical characteristics, and training only the final layers is more computationally efficient. ML and conventional statistical methods aim to enhance prediction accuracy by minimizing a loss function, such as the mean of squared errors. A high loss indicates that the model performed poorly, and a low loss indicates a good-fit model. Cross-validation is used in the modeling process to determine which model performs best while remaining robust to data not encountered during training. By sampling multiple pairs of training and test data from a limited data set, one can ensure that the performance goals are met and that the extent of training has been adequate while preventing over-fitting. There is no one-size-fits-all indicator for forecast accuracy. We have used Mean Squared Error (MSE), Root Mean Squared Error (RMSE), and Mean Absolute Percentage Error (MAPE) metrics for evaluation purposes across all four models to evaluate the goodness of predictions.

MSE is computed by squaring differences between the predicted and actual values and averaging the result. The range of MSE is between 0 and *∞*; the lower the MSE value, the more accurate the prediction model. MSE is the loss function of linear regression by default in ML. The MSE for our models can be expressed as:

(2) $$MSE_{M\_DNN}=\frac{1}{n}\sum_{i=1}^{n}{\left(y_{actual}-y_{predicted_{M\_DNN}}\right)}^{2}$$

(3) $$MSE_{ECO6G}=\frac{1}{n}\sum_{i=1}^{n}{\left(y_{actual}-y_{predicted_{ECO6G}}\right)}^{2}$$

(4) $$MSE_{ARIMA}=\frac{1}{n}\sum_{i=1}^{n}{\left(y_{actual}-y_{predicted_{ARIMA}}\right)}^{2}$$

(5) $$MSE_{ETS}=\frac{1}{n}\sum_{i=1}^{n}{\left(y_{actual}-y_{predicted_{ETS}}\right)}^{2}$$

MAPE is more robust than MSE to outliers in the dataset, and it expresses accuracy as a percentage of the error and measures the forecast error concerning actual values. The lower the MAPE value, the more accurately the ML model predicts values. MAPE less than a value of 10 percent indicates highly accurate forecasting. The MAPE for our models can be expressed as:

(6) $$MAPE_{M\_DNN}=\frac{1}{n}\sum_{i=1}^{n}|\frac{y_{actual}-y_{predicted_{M\_DNN}}}{y_{actual}}|*100$$

(7) $$MAPE_{ECO6G}=\frac{1}{n}\sum_{i=1}^{n}|\frac{y_{actual}-y_{predicted_{ECO6G}}}{y_{actual}}|*100$$

(8) $$MAPE_{ARIMA}=\frac{1}{n}\sum_{i=1}^{n}|\frac{y_{actual}-y_{predicted_{ARIMA}}}{y_{actual}}|*100$$

(9) $$MAPE_{ETS}=\frac{1}{n}\sum_{i=1}^{n}|\frac{y_{actual}-y_{predicted_{ETS}}}{y_{actual}}|*100$$

**Figure 2:**
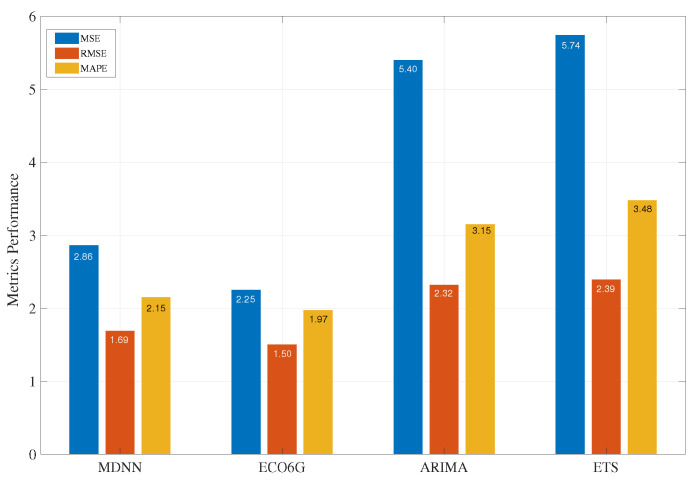
Model Evaluation and Metrics.

ARIMA is a time series analysis model that is fitted to time series data to better forecast future time series points. ARIMA uses trends, as well as cyclic, seasonal, and irregular changes, to characterize time features and sequences in patterns. Forecasting techniques based on ETS use a weighted sum of past observations, but the weights decrease exponentially. We have simulated network load for 168 h (about one week) using all models as a comparative study. Figure 2 shows the performance of all models in terms of MSE, RMSE, and MAPE metrics. Our proposed ECO6G algorithm performs better than the other three algorithms in error and accuracy metrics for the given dataset. In addition, our proposed algorithm has steady performance and converges faster because of pre-trained weights. Compared to the traditional neural network model ($M_{DNN}$), ECO6G yields 21% less error and 8.5 percent more accuracy for the given dataset.

**Figure 3:**
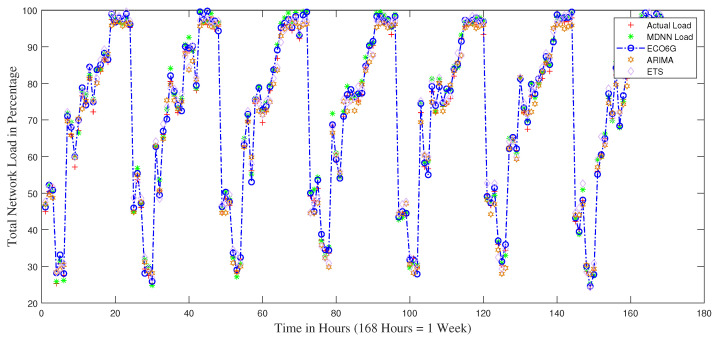
Simulation results of Network Load Prediction using Neural Network and Statistical Modeling.

Figure 3 demonstrates the simulated forecasted network load results from all models. The load change period is seven days and reflects the peak and off-hours variation through the day for a week, reflecting the real-world scenario. The figure also depicts the two prominent characteristics of mobile traffic and forecasting. First, the cell load is typically characterized by a strong periodicity, with periods of low load occurring from night to early morning. Second, the forecasting mechanism may produce non-negligible errors, meaning that deactivating cells at the incorrect time may significantly affect system performance. ECO6G closely follows the actual traffic load, which shows the model is reasonably accurate and provides reasonable confidence to use it against real-world network resource modeling. The difference between the average of all ECO6G estimates and the average of all actual values is only 1.10%, i.e., ECO6G is over-predicting by a small margin, which can be compensated against any spike in unusual traffic load to accommodate network resources during network planning.

Table 2 shows the simulated average and peak load values across all models. Daily average traffic over 24 h is modeled through three traffic loads (low, medium, and high) per ETSI ES 202 706-1 definition. For a weak validation, the ECO6G, $M_{DNN}$, and ETS models have predicted a positive delta (meaning the network would over-provision) in the case of average load against actual load for all three load scenarios. At the same time, ARIMA estimated a negative delta (under-provisioned). For a mobile network operator, it is moderately fair to over-provision to accommodate any spike in traffic but not by a large sum.

## 9. Experimentation Results of Cost–Benefit Analysis

Load-aware metrics are crucial for the next generation of green communication networks. One of the primary objectives of 5G networks for enhancing EE is to match system capacity and power consumption with network load. The total system EE for different load scenarios is defined in ETSI ES 202 706 and 3GPP TR 32.972 version 16.1.0 [6] using the following equations.

(10) $$EE_{global}=\sum_{low\;load}b_{low\;load}*EE_{low\;load}$$

(11) $$EE_{global}=\sum_{med\;load}b_{med\;load}*EE_{med\;load}$$

(12) $$EE_{global}=\sum_{high\;load}b_{high\;load}*EE_{high\;load}$$

EE (bits/joules) can be defined as the amount of traffic served per second by a BS (bits/s) divided by the power utilized by a BS to provide service (Watt = Joule/s) multiplied by a weighting factor *‘b’* based on the number of hours per day in each load condition. The ETSI TR [41] load levels are 10%, 30%, and 50% for low, medium, and high loads, respectively. This weighting factor *‘b’* takes on the value 6/24 for low load conditions in the last 6 h of a typical day: low load, 10/24 medium load, and 8/24 high load. With the EE equations defined in (10)–(12), the network EE can now be defined as the ability to minimize energy consumption relative to the provided traffic capacity. RAN EE is the measure of the capability of RAN elements to sustain a much better mobile broadband data rate while minimizing the BS energy consumption. The definition of RAN EE specified by the 3GPP is as follows:

(13) $$RAN_{EE}\left(\mathrm{bits}/\mathrm{joules}\right)=\frac{DataVolume}{Energy\;consumption}$$

where the unit of EE is bits/Joule, the unit for the data volume is bits/s/km${}^{2}$ and the unit of energy consumption is Joules/km${}^{2}$.

The typical and peak electrical power requirement for radio BSs (macro cell, micro cell, and pico or femtocell) related to aggregated RF power as defined in the ETSI ES 203 700 V1.1.1 [7] is used for the energy consumption calculation. In the case of a complex macro BS, the peak power consumption is $P_{max}$ = 24 kW, which includes multiple frequencies across 2G/3G/4G/5G radios and MIMO configuration, and the typical consumption is 8 kW. The bits per watt can be calculated for all three loads as follows:

(14) $$Bits\;per\;watts=\frac{Peak\;load_{low}}{P_{max}*P_{low\;load\;level}}*10^{6}$$

(15) $$Bits\;per\;watts=\frac{Peak\;load_{medium}}{P_{max}*P_{medium\;load\;level}}*10^{6}$$

(16) $$Bits\;per\;watts=\frac{Peak\;load_{high}}{P_{max}*P_{high\;load\;level}}*10^{6}$$

**Figure 4:**
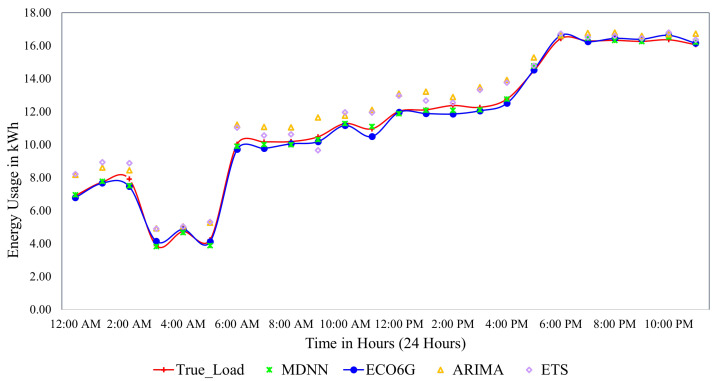
Base station’s typical daily energy usage.

Therefore, with respect to the power consumption vs. load (from [44]), the power consumption values are $P_{lowloadlevel}=0.46$, $P_{mediumloadlevel}=0.58$, and $P_{highloadlevel}=0.7$, respectively. Using Table 3, power consumption using the average load for a typical day across all load scenarios is calculated as shown in Table 4:

In most cases, as traffic volume and the number of utilized resources decreases, the energy consumed decreases linearly. Figure 4 depicts an analysis of typical energy consumption over a day by the BS based on the traffic pattern. The data analysis reveals that the difference between the minimum and maximum BS energy consumption is 4.15 kWh and 16.67 kWh for ECO6G model. The graph establishes the superiority of our ECO6G model, as it can achieve an improvement of 1.03% over the actual daily power consumption load for the given dataset.

Additionally, the average retail price per kilowatt-hour (kWh) in the US is USD 0.1177 for commercial uses, as of drafting this paper [53], so the OPEX cost to operate one BS for a day and for 5 years can be calculated as in Table 5. Note how close the ECO6G calculation is to Actual (within $4.31, only a 0.007% error).

## 10. Plausible ECO6G Use Cases in B5G Implementation

A developed country such as the United States of America has four major network operators. Suppose each operator deploys one hundred thousand 5G sites. In that case, the OPEX savings opportunity using the ECO6G load prediction model for weighted average load is close to 1.2 billion dollars over five years against other data-driven model predictions for each MNO. These savings will be in multiple if we consider global deployment from more than 750 MNOs deploying 5G, where 469 telecom operators from 140 countries/regions have already invested in 5G, while 182 telecoms from 73 countries/regions have started their own commercial 5G services [1].

We conducted five different experiments using test data and conclude that the ECO6G model predicted 100.57% better OPEX savings for low-load, medium-load, and high-load scenarios for the given dataset against other data-driven models and accurately predicting the network load. Thus, utilizing ECO6G, we can improve the OPEX saving for different load levels. As shown in Table 6, an approximate saving of 374 million dollars against $M_{DNN}$, 1422 million dollars against ARIMA, and approximately 1872 million dollars against ETS can be achieved by using ECO6G in all load scenarios considering 100,000 BSs over a five-year period.

With 5G rapidly expanding globally and more sophisticated 5G-Advanced features planned in 3GPP Release-18, industry, standards bodies, and research organizations are setting the groundwork for the next generation’s global sixth-generation (6G) communication standard. AI has the potential to become the foundation for the 6G air interface and network, making data, computing, and energy the new resources that can be used to achieve higher performance. As a result, 6G will have to deliver significantly more data at faster rates than current networks while also meeting extremely stringent EE goals to achieve a sustainable 6G system. This necessitates a significant reduction in the amount of energy needed to transmit a bit and the need for solutions that can be leveraged to attain energy-efficient next-generation networks.

The ECO6G model can be applied in multiple scenarios, such as enabling a 3GPP-compliant analytics service delivered in the form of statistics or predictions, intelligence operational in real time for Network Functions, Application functions (AFs), and operations, administration, and maintenance (OAM) services. The serving BS, for example, receives assistance data from RAN, such as load status, active UEs, QoS needs and energy consumption status [50]. The serving node executes an ML algorithm on the collected data to choose an energy-saving action that maximizes network efficiency while maintaining service quality. The node may announce its intention to offload traffic to neighboring nodes to conserve energy. Additionally, a single analytics source in an environment with multiple vendors, especially with Open-RAN (O-RAN) concepts, could be beneficial. We are currently investigating ECO6G use cases in the core and the edge locations with application-aware output for User plane function (UPF) load, especially with Multi-Access Edge Computing (MEC).

## 11. Conclusions

In this paper, we investigate traffic forecasting models to enable network management to enhance the 5G OPEX savings. The paper highlights the use of ML algorithms to predict the network load using network slicing KPIs and then uses the simulated predicted load to compute the OPEX savings per industry standards definition. We presented a comparative time-series study between neural network and statistical models and highlighted the proposed ECO6G superior metrics over other models. We are investigating the feasibility of ECO6G to supplement the 3GPP specified Network Data Analytics Function (NWDAF), introduced as part of Rel-16, which is intended to streamline how core network data is consumed to develop insights and take actions to improve the end-user experience. We firmly believe the ECO6G model can enable slice-level analytics and provide either statistics or forecasts of the performance of network load when used in conjunction with RAN systems, can be further used to design and orchestrate energy-efficient network planning.

## Figures and Tables

**Table 1:** Comparison of ECO6G against state-of-the-art methodologies.

Sr. No.	Related Work	ECO6G Work
[11]	Use of the cellular traffic types (SMS, phone and web), to train LSTM for slice resource allocation	Use of the network KPIs:RRC, RSSNI and PDU to train a DNN for predicting total load estimation
[12]	5G network slicing model using the DBN and NN to improve accuracy	TL-based DNN model for improving 5G energy efficiency and ensuring faster convergence
[13]	DDPG slice optimization and TL based multi agent DDPG (TMDDPG) for accelerated learning by evaluating delay, EE, and PLR for DDPG, DQN and TMDDPG	Evaluation of ARIMA, ETS, and DL models to investigate traffic forecasting for enhanced 5G EE
[14]	DRL based 5G RAN slicing resource allocation and TL to accelerate the learning and tackle slow convergence	Use of TL with DNN to estimate the network load using slicing KPIs, to estimate the EE and improved convergence rate
[15]	TL-based A2C approach to increase network utility at the expense of reduced adaptability of the various network topologies.	TL approach to improve energy efficiency with an approximate OPEX savings of seven hundred eighty-six million for the MNOs in off-peak network load scenarios
[16]	RAN slicing architecture for autonomous learning in interference affected and the TL approach to facilitate self-learning RAN slicing control.	The work in [16] targets autonomous RAN slicing, whereas our work uses the data driven model trained on the network KPIs to estimate the EE of 5G networks
[17]	Dynamic slicing resource allocation with an hourly dataset of a live cellular network attributes recorded over five days for sites in dense urban areas fed directly to the GRU	Our dataset is captured on a real-world 5G BS using the MNO’s proprietary software, including data for three sectors and network KPIs from each sector
[18]	Comparative analysis of the transfer RL (TRL), Q-value TRL and action selection TRL with model-free Q-learning and the model-based priority proportional fairness and time-to-live (PPF-TTL) to solve for slow convergence and lack of generalization of RL techniques	In contrast to [18], our work addresses the issue of slow convergence by proposing a comparative analysis of our ECO6G model with ARIMA, ETS, and DNN with random weights
[19]	Use of techniques for enabling sleep mode methods in heterogeneous mobile networks with the aim of reducing power consumption	Our work proposes to enhance the energy efficiency of the 5G network with an OPEX saving from the perspective of MNOs
[20]	EE DRL based resource allocation for RAN slicing to improve computational and time complexity	Data driven approach for improved OPEX savings against the conventional approaches for MNOS in varying load
[21]	DL based network slicing short-term traffic prediction for 5G transport network	Supervised ML model for forecasting traffic load and using the estimated load to evaluate EE and improve OPEX savings by a margin of 48.67% against other evaluated data-driven models

**Table 2:** Simulation results of average and peak load across all models.

**Average Load %**	**Low Load (6/24)**	**Medium Load (10/24)**	**High Load (8/24)**
Actual	42.53	74.36	88.23
$M_{DNN}$	43.1	75.65	89.04
ECO6G	43.03	75.21	88.96
ARIMA	42.01	73.92	87.63
ETS	43.6	75.4	88.84
**Peak Load %**	**Low Load (6/24)**	**Medium LOAD (10/24)**	**High LOAD (8/24)**
Actual	73.86	94.50	99.60
$M_{DNN}$	74.44	95.32	99.59
ECO6G	74.48	95.23	99.78
ARIMA	75.39	92.96	96.86
ETS	75.15	94.21	99.61

**Table 3:** Peak bits per watts calculation.

Peak Bits/Watts	Low Load (6/24)	Medium Load (10/24)	High Load (8/24)	Total
Actual	6690.22	6788.79	5928.57	6477.41
$M_{DNN}$	6742.75	6847.70	5927.98	6514.89
ECO6G	6746.38	6841.24	5939.29	6516.87
ARIMA	6828.80	6678.16	5765.48	6411.59
ETS	6807.07	6767.96	5929.17	6498.14

**Table 4:** Energy consumption for a typical day.

Power Consumption (in kW)	Low Load (6/24)	Medium Load (10/24)	High Load (8/24)	Total
Actual	6.36	10.95	14.88	11.11
$M_{DNN}$	6.39	11.05	15.02	11.21
ECO6G	6.38	10.99	14.98	11.17
ARIMA	6.15	11.07	15.20	11.22
ETS	6.41	11.14	14.98	11.24

**Table 5:** OPEX Cost (in $) for MNO to operate ‘a’ BS for 5 years.

OPEX Cost per BS ($)	Low Load (6/24)	Medium Load (10/24)	High Load (8/24)	Weighted Avg for 24 h
Actual	34,297.91	56,244.90	77,801.83	57,943.80
$M_{DNN}$	34,561.10	56,764.43	78,064.91	58,313.76
ECO6G	34,234.89	56,167.78	77,932.57	57,939.49
ARIMA	36,089.28	57,722.90	78,865.72	59,632.10
ETS	36,729.73	58,272.96	79,048.24	59,812.24

**Table 6:** OPEX Cost Change (in Million $) for MNO to operate ‘100,000’ BS for 5 year.

OPEX Cost Change across Models	Low Load (6/24)	Medium Load (10/24)	High Load (8/24)	Weighted Avg for 24 h (Also Compared with ECO6G)
$M_{DNN}$	263.19	519.53	263.09	369.96 (+374.27)
ECO6G	−63.02	−77.12	130.74	−4.31
ARIMA	1791.37	1478.00	1063.89	1418.30 (+1422.61)
ETS	2431.82	2028.06	1246.41	1868.45 (+1872.76)

## Data Availability

Not applicable.
